# A time-frequency feature fusion-based deep learning network for SSVEP frequency recognition

**DOI:** 10.3389/fnins.2025.1679451

**Published:** 2025-09-29

**Authors:** Yiwei Dai, Zhengkui Chen, Tian-ao Cao, Hongyou Zhou, Min Fang, Yanyun Dai, Lurong Jiang, Jijun Tong

**Affiliations:** ^1^School of Information Science and Engineering, Zhejiang Sci-Tech University, Hangzhou, China; ^2^School of Computer Science and Technology, Zhejiang Sci-Tech University, Hangzhou, China; ^3^School of Instrumentation Science and Engineering, Harbin Institute of Technology, Harbin, China; ^4^Weihai Sunfull Electronics Group Co., Ltd., Weihai, China; ^5^Zhejiang Key Laboratory of Research and Translation for Kidney Deficiency-Stasis-Turbidity Disease, Hangzhou, China

**Keywords:** steady-state visual evoked potentials, brain-computer interface, dual-feature extraction branch, convolutional neural network, feature fusion

## Abstract

**Introduction:**

Steady-state visual evoked potential (SSVEP) has emerged as a pivotal branch in brain-computer interfaces (BCIs) due to its high signal-to-noise ratio (SNR) and elevated information transfer rate (ITR). However, substantial inter-subject variability in electroencephalographic (EEG) signals poses a significant challenge to current SSVEP frequency recognition. In particular, it is difficult to achieve high cross-subject classification accuracy in calibration-free scenarios, and the classification performance heavily depends on extensive calibration data.

**Methods:**

To mitigate the reliance on large calibration datasets and enhance cross-subject generalization, we propose SSVEP time-frequency fusion network (SSVEP-TFFNet), an improved deep learning network fusing time-domain and frequency-domain features dynamically. The network comprises two parallel branches: a time-domain branch that ingests raw EEG signals and a frequency-domain branch that processes complex-spectrum features. The two branches extract the time-domain and frequency-domain features, respectively. Subsequently, these features are fused via a dynamic weighting mechanism and input to the classifier. This fusion strategy strengthens the feature expression ability and generalization across different subjects.

**Results:**

Cross-subject classification was conducted on publicly available 12-class and 40-class SSVEP datasets. We also compared SSVEP-TFFNet with traditional approaches and principal deep learning methods. Results demonstrate that SSVEP-TFFNet achieves an average classification accuracy of 89.72% on the 12-class dataset, surpassing the best baseline method by 1.83%. SSVEP-TFFNet achieves average classification accuracies of 72.11 and 82.50% (40-class datasets), outperforming the best controlled method by 7.40 and 6.89% separately.

**Discussion:**

The performance validates the efficacy of dynamic time-frequency feature fusion and our proposed method provides a new paradigm for calibration-free SSVEP-based BCI systems.

## 1 Introduction

The brain-computer interface (BCI) enables direct interaction between human beings and external devices by decoding Electroencephalogram (EEG) signals, conveying users’ intentions without peripheral nerves or muscles (Wolpaw et al., 2000). The applications of BCI covers assisting paralyzed patients in operating equipment, controlling wheelchairs or robotic arms, assembly of industrial products and intelligent home control (Dong et al., 2022). EEG is the principal source for noninvasive BCI systems on account of its low cost, portability, and high temporal resolution (Abiri et al., 2019). Steady-state visual evoked potential (SSVEP) (Nijboer et al., 2008), motor imagery (Ge et al., 2021) and P300 (Ang et al., 2008) are several common experimental BCI paradigms. In particular, SSVEP-based BCI has attracted significant attention due to the high information transfer rate, rich command set, and minimal training requirements (Zhang et al., 2022; Rostami et al., 2022), bringing promising applications in smart home control (Chai et al., 2020), clinical rehabilitation (Wang et al., 2023), and assistive communication (Rezeika et al., 2018). Researchers believe that various neural networks distributed in the brain have their inherent resonant frequencies. Under resting state, these neural networks are all asynchronous with each other and are disordered, without any regularity. At this time, the EEG signals are spontaneous brainwaves. When a constant-frequency external visual stimulus is applied, the neural networks that are in phase with the stimulus frequency or its harmonics will resonate, resulting in significant sustained oscillatory response in the brain’s potential activity at the stimulus frequency and its harmonics, thereby generating the SSVEP signal (Zhou, 2008; Wang et al., 2020).

In SSVEP-based BCI systems, the primary task is to decode the user’s intention accurately by identifying the frequency of the attended visual stimulus through EEG processing (Liu et al., 2022). To enhance the reliability of SSVEP-BCI systems, a variety of frequency-recognition methods have been developed, spanning from traditional signal processing techniques to current deep learning approaches. However, most methods focus on single domain features, which decreases the recognition rate and information transfer rate (ITR). Additionally, there is a lack of model generalization in cross-subject scenarios. In this case, we propose the SSVEP time-frequency fusion network (SSVEP-TFFNet) for SSVEP frequency recognition, an improved deep learning network fusing time-domain and frequency-domain features dynamically. The network consists of two parallel branches: a time-domain branch that ingests raw EEG signals and a frequency-domain branch that processes complex-spectrum features. The two branches extract the time-domain and frequency-domain features separately. These features are fused via a dynamic weighting mechanism afterwards and input to the classifier.

The remainder of this paper is as follows: Section 2 reviews the related work. Section 3 describes the overall schematic and relevant theories, including the datasets and our proposed SSVEP-TFFNet model. Section 4 lists the results step by step. Section 5 discusses the effect of channel number, ablation analyses and model interpretability. Section 6 summarizes the full text.

## 2 Literature review

### 2.1 Traditional methods in SSVEP frequency-recognition

Early SSVEP frequency-recognition methods relied on fast Fourier transform (FFT) to convert EEG signals from time domain into frequency domain, identifying the stimulus frequency by detecting the spectral peak on a single EEG channel. However, this approach is highly susceptible to noise and requires relatively long time windows to achieve acceptable accuracy (Cheng et al., 2002). Subsequently, canonical correlation analysis (CCA) was introduced and widely utilized. CCA synthesizes reference sinusoidal signals at each candidate stimulus frequency, and computes the canonical correlation coefficients between multichannel EEG signals and each reference signal. The stimulus frequency with the highest canonical correlation coefficient is selected as the predicted frequency (Lin et al., 2006). Apart from the fundamental frequency in SSVEP, harmonics can provide additional discriminative information. Filter bank CCA (FBCCA) was then introduced to decomposes the SSVEP into multiple sub-bands via a bank of bandpass filters and fuses fundamental and harmonic components afterwards to improve frequency detection performance (Chen et al., 2015). Later, task-related component analysis (TRCA) for SSVEP frequency recognition was adapted for the first time (Nakanishi et al., 2017). TRCA used each subject’s EEG as a template and maximized the covariance between trials to derive spatial filters that extracted task-related components. The classification accuracy achieved up to 89.83% in SSVEP-BCI systems. However, TRCA tends to produce redundant spatial filters for each stimulus and is not capable of fully exploiting temporal information. Accordingly, task-discriminant component analysis (TDCA) was proposed to further improve the performance under individual calibration (Liu et al., 2021). However, these traditional methods are constrained by relying on single domain feature, which limits the capacity to capture high-level features. Consequently, during the classification of complex EEG signals, both the classification accuracy and the ITR are relatively low (Lei et al., 2024). Especially, in cross-subject scenarios, substantial inter-subject variation in EEG characteristics leads to dramatic degradation of classification performance. Robust cross-subject SSVEP classification is critical for practical BCI deployment.

### 2.2 Deep learning methods in SSVEP frequency-recognition

Over the past decade, deep learning have achieved significant progress in biosignal analysis (LeCun et al., 2015). Because of the ability to learn representations in an end-to-end manner, deep neural networks have been gradually applied to EEG analysis in recent years (Roy et al., 2019; Lawhern et al., 2018). Regarding the characteristics of SSVEP signals, researchers have designed various neural networks.

Time domain features are usually extracted in SSVEP analysis. EEGNet is a compact convolutional neural network (CNN) that employed depthwise separable convolutions to automatically extract discriminative features from multichannel SSVEP time-domain signals (Waytowich et al., 2018). EEGNet required no subject-specific calibration and demonstrated superior cross-subject adaptability compared to traditional methods. Time-domain-based CNN (tCNN) was proposed to model the temporal dynamics of SSVEP signals via one-dimensional time-domain convolutions (Ding et al., 2021). Later, the filter bank tCNN (FB-tCNN) was further proposed. FB-tCNN could process multiple band-pass sub-bands in parallel and fuse their discriminative information, making it effective for frequency recognition especially with short time windows. SSVEPNet integrated one-dimensional convolutions with a long short-term memory (LSTM) network to enhance temporal modeling (Pan et al., 2022). This model incorporated spectral normalization and label-smoothing regularization to mitigate overfitting, achieving the classification accuracies of 84.45 and 84.22% on the four-class and twelve-class datasets.

Apart from time domain features, frequency domain features are taken advantage of in SSVEP analysis as well. CNN trained on complex spectrum features (C-CNN) was designed (Ravi et al., 2020). It is a shallow convolutional network focused on frequency-domain feature extraction. The raw EEG signals were first transformed by FFT, and the resulting real and imaginary components were concatenated as network input. Thence, both amplitude and phase were captured and rich spectral details were maintained with low computational cost.

Deep learning methods utilizing features from different domains are gradually made use of for SSVEP recognition. An effective data-augmentation technique called EEG mask encoding (EEG-ME) was introduced to mitigate overfitting (Ding et al., 2024). EEG-ME masked portions of the EEG so as to encourage the network to learn more robust features and improve generalization. In order to model the spatial-topological structure of EEG signals more effectively, a network integrated a temporal feature extractor, a spatial topology converter and a multigraph subspace module (TSMNet) was presented for SSVEP classification (Deng et al., 2025). The proposed model realized the classification accuracies of 84.76 and 73.95% on two publicly available datasets.

Although these deep learning approaches have made progress to some extent, undeniable inter-subject variability in EEG signals, stemming from factors such as age, sex, and lifestyle (Liu et al., 2024; Huang et al., 2023), still provokes low classification accuracy and poor generalization in calibration-free and cross-subject scenarios. A higher accuracy usually depends on collecting subject-specific calibration data to train the models. However, EEG acquisition is usually laborious and time-consuming, limiting the practical applicability of SSVEP-BCI systems (Chiang et al., 2019). Meantime, current methods tend to learn features exclusively in either time domain or frequency domain. Time-domain based approaches focus on capturing temporal dynamics but may fail to extract stable spectral characteristics. Frequency-domain based methods utilize only static spectral information and overlook time-varying properties of signal (Singh and Krishnan, 2023).

To achieve robust performance across different subjects, there is a need to extract effective features and transfer learned recognition patterns to new users. In this paper, we propose the SSVEP time-frequency fusion network (SSVEP-TFFNet) for SSVEP frequency recognition. SSVEP-TFFNet comprises two parallel feature-extraction branches: a time-domain branch that processes raw EEG signals and a frequency-domain branch that operates on complex-spectrum features. Outputs from both branches are fused via a dynamic weighting mechanism. The fused features are fed into two fully connected layers to perform frequency classification.

## 3 Materials and methods

### 3.1 Experimental paradigm and preprocessing

Dataset A, 12JFPM (Nakanishi et al., 2015), comprises EEG signals recorded from 10 subjects with normal or corrected-to-normal vision. Each subject was exposed to 12 distinct visual-stimulus frequencies. Stimuli were displayed on a 27-inch LCD monitor (60 Hz refresh rate) arranged in a 4 × 3 grid. Frequencies ranged from 9.25 Hz to 14.75 Hz in 0.50 Hz increments, and phases were initialized at 0 and increased by 0.5π per stimulus. Each subject completed 15 sessions and each session contained 12 trials presented in random order, i.e., one trial per target frequency. When each trial began, a red square appeared at the target location for 1 s, during which subjects were instructed to fixate on the target. Subsequently, all stimuli flashed for 4 s simultaneously. Subjects were asked to minimize eye blinks during this interval to reduce Electrooculogram (EOG) artifacts. EEG signals were recorded using a BioSemi ActiveTwo system at 2048 Hz via eight Ag/AgCl electrodes positioned over the occipital region (PO7, PO3, POz, PO4, PO8, O1, Oz, and O2). All data were downsampled to 256 Hz. A fourth-order Butterworth bandpass filter between 6 Hz and 80 Hz was made use of to reserve the effective component of SSVEP. Given the visual-evoked latency, epochs were extracted 0.135 s after the stimulus began.

Dataset B, BETA (Liu et al., 2020), consists of EEG signals recorded from 70 healthy subjects exposed to 40 distinct visual-stimulus frequencies. Stimuli were arranged in a keyboard-like layout and presented on a 27-inch LED monitor with a 60 Hz refresh rate. Frequencies ranged from 8 Hz to 15.8 Hz in 0.2 Hz increments and phases were initialized at 0 and advanced by 0.5 π for each frequency. Each subject completed four sessions, each comprising 40 trials in which the 40 target stimuli were presented in random order. Each trial began with a 0.5 s visual cue, followed by synchronous flashing of all targets. The flashing lasted 2 s for the first 15 subjects and 3 s for the remaining 55 subjects. The trail ended with a 0.5 s rest. EEG data were acquired with a SynAmps2 system at 1,000 Hz from 64 channels configured according to the international 10–10 system. A built-in notch filter removed 50 Hz power frequency interference, and signals were subsequently downsampled to 250 Hz. Unlike Dataset A, all EEG signals in Dataset B were collected in a non-shielded environment to reflect real-world conditions. The computational cost of leave-one-subject-out cross-validation (LOSOCV) increases rapidly as the number of subjects grows. As a result, expanding the number of subjects not only requires an enhancement in the total number of validated subjects, but also doubles the training time for each subject, which prolongs the experimental period significantly. Taking other research (Ravi et al., 2020; Pan et al., 2022) into consideration as well, we eventually chose 35 subjects in the analysis. In the meantime, to avoid posterior bias caused by subjective selection, we adopted a deterministic and performance independent selection rule: The top 35 subjects were selected based on the original index of the dataset, rather than screening based on results or individual characteristics. In this paper, EEG signals from nine electrodes over the parieto-occipital region (Pz, PO3, PO5, PO4, PO6, POz, O1, Oz, and O2) were analyzed. Preprocessing comprised a fourth-order Butterworth bandpass filter between 7 Hz and 64 Hz, and epochs were extracted 0.13 s after stimulus started.

Dataset C, Benchmark (Wang et al., 2017), gathers SSVEP-BCI recordings of 35 healthy subjects focusing on 40 characters flickering at different frequencies (8–15.8 Hz with an interval of 0.2 Hz). For each subject, the experiment consisted of 6 blocks. Each block contained 40 trials corresponding to all 40 characters indicated in a random order. Each trial started with a visual cue (a red square) indicating a target stimulus. The cue appeared for 0.5 s on the screen. Subjects were asked to shift their gaze to the target as soon as possible within the cue duration. Following the cue offset, all stimuli started to flicker on the screen concurrently and lasted 5 s. After stimulus offset, the screen was blank for 0.5 s before the next trial began, which allowed the subjects to have short breaks between consecutive trials. Each trial lasted a total of 6 s. To facilitate visual fixation, a red triangle appeared below the flickering target during the stimulation period. In each block, subjects were asked to avoid eye blinks during the stimulation period. To avoid visual fatigue, there was a rest for several minutes between two consecutive blocks. EEG data were acquired with a sampling rate of 1,000 Hz. The amplifier frequency passband ranged from 0.15 Hz to 200 Hz. Sixty-four channels covered the whole scalp of the subject and were aligned according to the international 10–20 system. To remove the common power-line noise, a notch filter at 50 Hz was applied in data recording. A fourth-order Butterworth bandpass filter between 7 Hz and 64 Hz was utilized as well. Event triggers generated by the computer to the amplifier and recorded on an event channel synchronized to the EEG data. The continuous EEG data was segmented into 6 s epochs (500 ms pre-stimulus, 5.5 s post-stimulus onset). The epochs were subsequently downsampled to 250 Hz. Similarly, EEG signals from nine electrodes over the parieto-occipital region (Pz, PO3, PO5, PO4, PO6, POz, O1, Oz, and O2) were analyzed.

### 3.2 The proposed module

**Figure 1:**
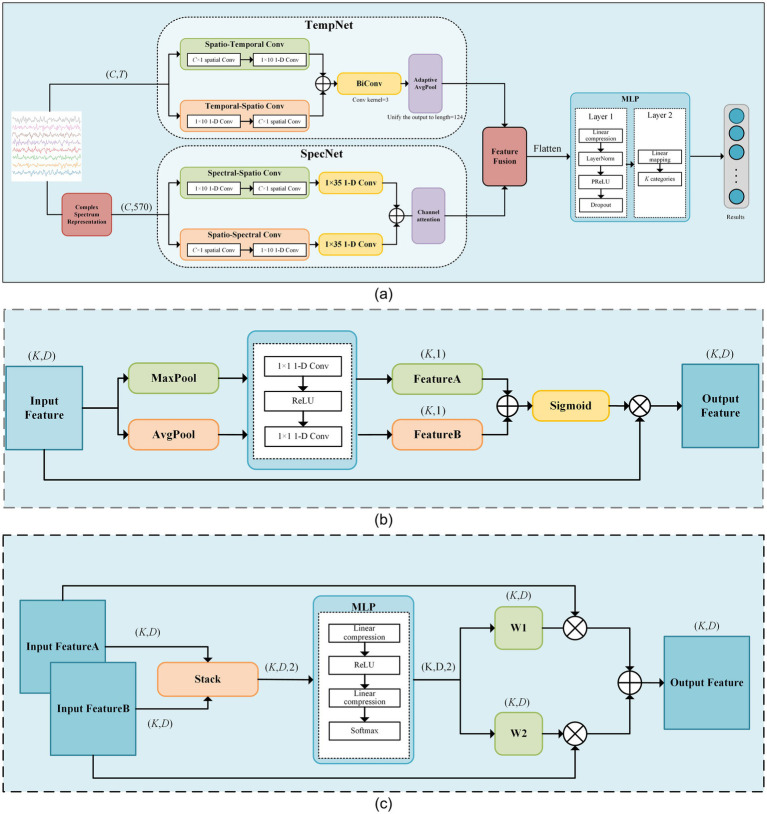
The framework diagram of our proposed model. **(a)** The schematic diagram of SSVEP-TFFNet. **(b)** Network of the channel-attention module. **(c)** Network of the feature fusion module.

The proposed SSVEP-TFFNet model consists of following parts: input module, time domain feature extraction branch (Temporal Net, TempNet), frequency domain feature extraction branch (Spectral Net, SpecNet), and feature fusion and classification modules, as displayed in Figure 1. Through the dual-feature extraction branch, the model is able to capture the complementary features of time domain and frequency domain from SSVEP signals efficiently, and fuse them adaptively to improve the classification accuracy.

#### 3.2.1 Input module

For time-domain branch, we input the preprocessed EEG signals directly. For frequency-domain branch, we first apply FFT to transform the time-domain signals into frequency domain signals. FFT can be expressed as Equation 1:


(1) FFT(x)=Re[FFT(x)]+jIm[FFT(x)]

where *x* denotes the preprocessed time-domain data, and j is the imaginary unit. Re and Im represent the real and imaginary parts of the FFT result, respectively. For the frequency-domain data, there are two approaches to convert it into model input, namely the magnitude spectrum $X_{\mathrm{mag}}$ and the complex spectrum $X_{\text{comp}}$ (Ravi et al., 2020), as shown in Equations 2 and 3:


(2) Xmag={Re[FFT(x)]}2+{Im[FFT(x)]}2

(3) Xcomp=Re[FFT(x)]‖Im[FFT(x)]

where the magnitude spectrum $X_{\mathrm{mag}}$ is computed as the sum of squares of the real and imaginary parts at each frequency point, considering the amplitude only and ignoring phase. In comparison, the complex spectrum $X_{\text{comp}}$ concatenates the real and imaginary parts, containing both amplitude and phase information. Early studies have suggest that phase information plays an important role in decoding SSVEPs (Pan et al., 2011). Therefore, we took $X_{\text{comp}}$ as the input to the frequency-domain feature extraction branch. The input to the frequency-domain branch, $I_{\text{spec}}$ , can be defined as Equation 4:


(4) Ispec=[Re[FFT(xCH1)],Im[FFT(xCH1)]Re[FFT(xCH2)],Im[FFT(xCH2)]Re[FFT(xCH3)],Im[FFT(xCH3)]...Re[FFT(xCHn)],Im[FFT(xCHn)]]

where $x_{\mathrm{CH}1}$ , $x_{\mathrm{CH}2}$ , $x_{\mathrm{CH}3}$ , $x_{\mathrm{CHn}}$ mean the EEG data from different channels, and *n* is the number of channels. In this study, the frequency resolution of the FFT was fixed at 0.2 Hz. We extracted the real and imaginary parts of frequency components from each channel between 7 Hz and 64 Hz, resulting in two vectors of length 285. These two vectors were concatenated into a feature vector of length 570. Thence, $I_{\text{spec}}$ could be viewed as a matrix of size CHn × 570.

#### 3.2.2 TempNet module

In TempNet, we adopted parallel convolutional paths to extract time-spatial cooperative features. In the spatial–temporal path, a *C* × 1 spatial convolution was first applied along the channel dimension, followed by a 1 × 10 one-dimensional convolution along the temporal dimension. *C* represents the number of channels of the EEG signals. In contrast, the temporal–spatial path applied a 1 × 10 convolution along the temporal dimension first, and then performed a *C* × 1 spatial convolution. Each convolution operation was followed by BatchNorm2d and PReLU activation, and Dropout was added at the end of each path to suppress overfitting. The outputs of the two paths were summed to achieve an initial fusion of temporal features, which were then fed into a bidirectional one-dimensional convolution layer that applied convolution kernels of size 3 in both forward and backward directions. The results of both directions were summed and passed through a 1 × 1 convolution to generate the final temporal features. Finally, AdaptiveAvgPool was taken advantage of adjust the dimensions of temporal features to match that of the frequency-domain features for subsequent fusion. The detailed parameters of TempNet are listed in Table 1.

**Table 1:** Parameters of TempNet.

Layer	Layertype	Kernal	Stride	Out	Shape	Options
Input				1	(*C*, *T*)	
Spatio-Temporal Conv	Conv2d	(*C*,1)	(1,1)	16	(1, T)	BtachNorm2d → PReLU
	Conv2d	(1,10)	(1,2)	32	(1, [(*T*-10)/2] + 1)	BtachNorm2d → PReLU
	Dropout					dropout rate = 0.5
Temporal-Spatio Conv	Conv2d	(1,10)	(1,2)	16	(*C*, [(*T*-10)/2] + 1)	BtachNorm2d → PReLU
	Conv2d	(*C*,1)	(1,1)	32	(1, [(*T*-10)/2] + 1)	BtachNorm2d → PReLU
	Dropout					dropout rate = 0.5
Squeeze				32	([(*T*-10)/2] + 1)	
BiCNN	Conv1d	3	1	32	([(*T*-10)/2] + 1)	padding = 1
	Conv1d	3	1	32	([(*T*-10)/2] + 1)	padding = 1, Reverse
	Conv1d	1	1	32	([(*T*-10)/2] + 1)	BatchNorm1d → PReLU
	Dropout					dropout rate = 0.5
AdaptiveAvgPool				32	(124)	

#### 3.2.3 SpecNet module

In SpecNet, we also designed two parallel convolutional paths to fully dig frequency-spatial cooperative features. The spectral-spatial path first applied a 1 × 10 one-dimensional convolution along the spectral dimension to extract local fine-grained frequency features. Next, it used a *C* × 1 spatial convolution to fuse information across channels. The spatial-spectral path first conducted a *C* × 1 spatial convolution along the channel dimension to capture inter-channel cooperative information, and then applied a 1 × 10 one-dimensional convolution along the spectral dimension to further refine the fused spatial features. Subsequently, both paths employed a 1 × 35 one-dimensional convolution to capture larger-scale spectral context, and their outputs were summed to form the integrated frequency-domain representation. Each convolutional operation was immediately followed by BatchNorm2d and PReLU activation, with dropout applied at the end of each path. To enhance the model’s ability to discriminate key frequency bands, a channel-attention module was introduced on the fused output. It first obtained channel descriptors via global average pooling and global max pooling. Then, it used two 1 × 1 convolutions and a Sigmoid mapping to dynamically weight each channel, thus improving the reliability of effective feature representations. The detailed parameters of SpecNet are given in Table 2.

**Table 2:** Parameters of SpecNet.

Layer	Layertype	Kernal	Stride	Out	Shape	Options
Input				1	(*C*,570)	
Spatio-Spectral Conv	Conv2d	(*C*,1)	(1,1)	16	(1,570)	BtachNorm2d → PReLU
	Conv2d	(1,10)	(1,2)	32	(1,281)	BtachNorm2d → PReLU
	Conv2d	(1,35)	(1,2)	32	(1,124)	BtachNorm2d → PReLU
	Dropout					dropout rate = 0.5
Spectral-Spatio Conv	Conv2d	(1,10)	(1,2)	16	(*C*,281)	BtachNorm2d → PReLU
	Conv2d	(*C*,1)	(1,1)	32	(1,281)	BtachNorm2d → PReLU
	Conv2d	(1,35)	(1,2)	32	(1,124)	BtachNorm2d → PReLU
	Dropout					dropout rate = 0.5
Channel attention				32	(1,124)	
Squeeze				32	(124)	

#### 3.2.4 Feature fusion and classification

During feature fusion, we first computed attention weights at each position of the time and frequency domain features using two fully connected layers followed by a Softmax function. These attention weights were then utilized to perform a weighted summation of the time and frequency domain features. The fused features were flattened and passed through a two-layer fully connected classification network. The first layer compressed the high-dimensional features into a lower-dimensional space linearly, followed by LayerNorm, PReLU activation, and Dropout to enhance expression capacity and suppress overfitting. The second layer mapped the hidden representation linearly to a *U*-dimensional category space. Through normalization and regularization, this design preserved nonlinear expressiveness while improving training stability and model generalization. The detailed parameters are given in Table 3.

**Table 3:** Parameters of the feature fusion and classification.

Structure	Layer	Kernal	Stride	Out	Shape	Options
Feature fusion				32	(124)	
MLP	Flatten			3,968		
	Linear			198		LayerNorm→PReLU
	Dropout					dropout rate = 0.5
	Linear			*K*		

We chose LOSOCV on the public datasets to evaluate the cross-subject generalization ability of the model. Specifically, in each round of experiments, the data from one subject were taken as the test set, and the data from the other subjects were selected for training, until the data of each subject were used once as the test set. All deep learning models were implemented in PyTorch. Previous studies (Pan et al., 2022; Miyato et al., 2018) have shown that spectral normalization helps improve the performance of SSVEP model. Therefore, we introduced this regularization technique into our proposed model. In our network, spectral normalization was applied to each convolutional layer and fully connected layer. Specifically, to enforce K-Lipschitz continuity on the weight matrix *W*, the minimum of *K* is $\sigma (W)=\sqrt{\lambda_{1}}$ , where $\lambda_{1}$ is on behalf of the largest singular of $W^{T}W$ . Thus, aiming at constraining W to satisfy 1-Lipschitz continuity and stabilize the network training process, we adjusted all elements of W as Equation 5:


(5) $${\bar{W}}_{\mathrm{SN}}(W)=\frac{W}{\sigma (W)}$$

In this study, we did not conduct a large-scale hyperparameter search. Instead, we adopted parameter settings based on experience and common practices to ensure a stable training process and the completion of all comparative experiments within a reasonable time. In other words, the parameter selection was “experience-driven” rather than the result of fine-tuning. This avoids masking the inherent advantages and disadvantages of the methods due to excessive parameter tuning and is more in line with the practical application scenarios. During training, the cross-entropy loss function was utilized and the optimizer was Adam. The initial learning rate was 0.001, the dropout rate was set to 0.5, and the number of epochs was 150. For Dataset A, the L2 regularization coefficient was set to 0.0001 and the batch size was set to 32. For Dataset B, the L2 regularization coefficient was 0.001 and the batch size was 128.

We made use of different batch sizes and L2 regularization coefficients for Dataset A and Dataset B mainly on account of the differences between two datasets: Dataset A has smaller scale and lower noise. Accordingly, a smaller batch size and weaker L2 were adopted to ensure the model has sufficient update flexibility. Dataset B and C have a larger sample size, more classes, and higher noise levels. Thus, a larger batch size was used to stabilize gradient estimation, and a stronger L2 was applied to suppress overfitting. This differentiated configuration is not the result of individual dataset-specific tuning but a reasonable empirical setting based on the scale and characteristics of the datasets. We fixed a set of uniform parameters for each dataset and kept them unchanged in all leave-one-subject-out experiments on one dataset, without adjusting them for individuals or specific experimental conditions. This ensures the fairness of the comparison.

Model performance is evaluated by classification accuracy and ITR. Accuracy is defined as Equation 6:


(6) $$P=\frac{l}{m}$$

where *l* means the number of correctly classified samples and *m* denotes the total number of samples. ITR measures the system efficiency. It accounts not only for accuracy but also for recognition speed and the number of classes. ITR (bits/min) is calculated as Equation 7, reflecting the information the BCI can transmit per second (Wolpaw et al., 2002).


(7) $$\mathrm{ITR}=[\log G2+P\log_{2}P+(1-P)\log_{2}\frac{1-P}{G-1}]\times \frac{60}{T}$$

where *G* is the number of stimulus targets, *P* means the accuracy, and *T* represents the length of time window. A high ITR indicates that the system delivers faster response speed while maintaining accuracy, which is critical for practical BCI applications.

## 4 Results

To validate the effectiveness of our proposed method, we compared it with other principal methods: FBCCA, TRCA, TDCA, EEGNet, CCNN, FBtCNN, and SSVEPNet. All methods were evaluated on Dataset A and B, and the average classification accuracy contained mean ± standard deviation.

### 4.1 Results on Dataset A

The LOSOCV was employed to evaluate the performance of each method under different time windows. Tables 4, 5 give the average classification accuracy and ITR for five time windows (epochs): 0.4 s, 0.6 s, 0.8 s, 1.0 s, and 1.2 s. As the time window length increases, the classification accuracy of all methods improves, as longer windows accumulate more SSVEP information. In contrast, shorter time windows provoke lower SNRs, making feature extraction more challenging. Meanwhile, ITR does not increase linearly with accuracy improvements. ITR is jointly influenced by accuracy and time window length. Results illustrate that SSVEP-TFFNet outperforms all controlled approaches across all time windows. Specifically, under the 0.4 s time window, our method achieves an average accuracy of 59.11%, outperforming TDCA (47.17%), EEGNet (56.06%), FBtCNN (50.56%) and SSVEPNet (55.67%) by 11.94, 3.05, 8.55 and 3.44%, respectively. As the time window increases from 0.4 s to 1.2 s, the accuracy of our method rises from 59.11 to 89.72%. Under the longest window (1.2 s), our method surpasses CCNN (85.22%) by 4.50%, TDCA (80.56%) by 9.16%, classical TRCA (81.17%) by 8.55%, and the FBtCNN (80.94%) by 8.78%. In terms of ITR, SSVEP-TFFNet also achieves the best performance across all time windows. Particularly, under the shortest window (0.4 s), our method reaches the highest ITR of 197.52 bits/min, exceeding SSVEPNet (178.42 bits/min), TDCA (132.13 bits/min) and FBtCNN (144.73 bits/min) by 19.10 bits/min, 65.39 bits/min and 52.79 bits/min separately. As the time window extends to 0.6 s and 0.8 s, although the accuracy grows to 68.89 and 79.61%, the ITR decreases to 177.94 bits/min and 174.36 bits/min. When extending the window to 1.0 s and 1.2 s, the ITR further drops down to 159.61 bits/min and 144.59 bits/min, respectively.

**Table 4:** Mean classification accuracy (%) across subjects for different methods under different time window lengths on Dataset A.

Method	Length of time (s)				
	0.4	0.6	0.8	1	1.2
FBCCA	17.44 ± 6.24	29.89 ± 12.35	44.56 ± 18.53	59.39 ± 22.50	67.17 ± 23.28
TRCA	49.17 ± 20.32	60.44 ± 24.45	69.33 ± 26.49	75.50 ± 26.48	81.17 ± 21.86
TDCA	47.17 ± 19.80	56.83 ± 24.64	66.89 ± 25.22	75.94 ± 23.77	80.56 ± 20.42
EEGNet	56.06 ± 19.32	65.50 ± 20.44	74.89 ± 19.36	80.39 ± 18.10	85.67 ± 15.21
CCNN	52.28 ± 17.28	63.17 ± 21.32	75.06 ± 21.32	81.22 ± 19.80	85.22 ± 17.43
FBtCNN	50.56 ± 17.26	61.00 ± 21.75	70.72 ± 22.45	76.50 ± 22.75	80.94 ± 21.00
SSVEPNet	55.67 ± 20.43	68.22 ± 22.85	76.44 ± 22.46	82.83 ± 19.65	87.89 ± 15.61
Ours	59.11 ± 19.76	68.89 ± 22.03	79.61 ± 20.38	85.39 ± 17.90	89.72 ± 13.74

**Table 5:** Mean ITR (bits/min) across subjects for different methods under varying time window lengths on Dataset A.

Method	Length of time (s)				
	0.4	0.6	0.8	1	1.2
FBCCA	12.08 ± 12.36	33.24 ± 29.67	58.52 ± 46.33	81.99 ± 52.73	86.13 ± 49.85
TRCA	143.04 ± 89.67	143.51 ± 83.87	140.32 ± 79.66	132.01 ± 70.37	122.63 ± 54.93
TDCA	132.13 ± 85.28	129.19 ± 82.20	129.88 ± 74.52	130.38 ± 65.38	119.29 ± 51.32
EEGNet	178.26 ± 104.49	159.66 ± 81.70	153.59 ± 68.74	140.31 ± 55.38	131.39 ± 42.81
CCNN	153.90 ± 83.51	150.46 ± 80.93	156.07 ± 71.80	144.91 ± 59.49	131.79 ± 47.55
FBtCNN	144.73 ± 82.95	141.87 ± 79.65	140.42 ± 70.51	131.20 ± 63.43	121.12 ± 53.07
SSVEPNet	178.42 ± 108.74	175.93 ± 93.50	163.47 ± 77.82	150.85 ± 60.86	139.50 ± 45.11
Ours	197.52 ± 110.75	177.94 ± 92.16	174.36 ± 72.77	159.61 ± 59.05	144.59 ± 41.46

### 4.2 Results on Dataset B

Similarly, we took advantage of LOSOCV. Since Dataset B was collected in a non-electromagnetically shielded environment, the noise level is substantially higher than that of Dataset A. We conducted preliminary experiments within a time window of 0.6 s, and raised the window length longer (0.8 s, 1.0 s, and 1.2 s). Tables 6, 7 manifest the classification accuracy and ITR of each method under different time windows. It is clear that all methods (including our proposed method and controlled method) had dramatically lower classification accuracies under 0.6 s time window. Considering the significant decrease in accuracy caused by short windows and the fact that the overall information transmission rate is not better than that under 0.8 s window, the results under shorter window has limited practical applications. As a consequence, we finally focused on time windows of 0.8 s and above in subsequent experiments.

**Table 6:** Mean classification accuracy (%) across subjects for different methods under different time window lengths on Dataset B.

Method	Length of time (s)			
	0.6	0.8	1.0	1.2
FBCCA	25.54 ± 10.53	40.07 ± 15.28	52.88 ± 18.12	61.84 ± 18.45
TRCA	28.48 ± 17.20	38.21 ± 20.44	44.00 ± 22.90	47.93 ± 23.89
TDCA	38.82 ± 18.83	46.79 ± 21.28	52.09 ± 22.55	56.64 ± 22.96
EEGNet	40.12 ± 20.29	49.93 ± 22.78	56.79 ± 22.98	62.52 ± 22.80
CCNN	38.09 ± 18.02	49.16 ± 21.53	57.55 ± 22.82	64.71 ± 22.34
FBtCNN	37.57 ± 18.59	45.23 ± 20.48	50.95 ± 21.54	55.52 ± 22.58
SSVEPNet	37.09 ± 19.12	46.71 ± 20.89	54.86 ± 22.53	60.62 ± 22.99
Ours	45.82 ± 19.33	56.62 ± 21.95	65.73 ± 22.84	72.11 ± 21.92

**Table 7:** Mean ITR (bits/min) across subjects for different methods under varying time window lengths on Dataset B.

Method	Length of time (s)			
	0.6	0.8	1.0	1.2
FBCCA	60.93 ± 40.58	94.27 ± 53.97	116.14 ± 58.96	122.79 ± 53.83
TRCA	78.67 ± 75.84	92.47 ± 76.04	92.51 ± 71.25	87.76 ± 64.42
TDCA	123.92 ± 90.67	124.19 ± 83.16	117.24 ± 73.36	110.71 ± 64.55
EEGNet	131.78 ± 101.81	138.10 ± 90.48	133.30 ± 76.50	127.90 ± 64.81
CCNN	119.65 ± 83.29	133.47 ± 85.56	135.73 ± 76.45	134.42 ± 66.15
FBtCNN	117.99 ± 88.06	117.60 ± 78.17	112.63 ± 69.51	107.17 ± 61.77
SSVEPNet	116.49 ± 91.85	123.46 ± 82.15	126.26 ± 74.75	122.33 ± 65.79
Ours	157.94 ± 98.75	164.75 ± 90.65	165.59 ± 80.32	158.60 ± 68.56

SSVEP-TFFNet performs best across all time windows. Under the 0.8 s time window, our method achieves an accuracy of 56.62% which is 9.83, 9.91 and 11.39% higher than TDCA (46.79%), SSVEPNet (46.71%) and FB-tCNN (45.23%), respectively. As the window increases to 1.0 s and 1.2 s, the accuracy rises to 65.73 and 72.11% further. Explicitly, under the 1.2 s time window, our method surpasses CCNN (64.71%) by 7.40%, TDCA (56.64%) by 15.47%, FBtCNN (55.52%) by 16.59%, and EEGNet (62.52%) by 9.59%, revealing better performance. In the light of ITR, our method performs best as well across all time windows. For the 0.8 s and 1.0 s windows, the ITR reaches 164.75 bits/min and 165.59 bits/min, exceeding TDCA (124.19 bits/min and 117.24 bits/min) by 40.56 bits/min and 48.35 bits/min, surpassing FBtCNN (117.60 bits/min and 112.63 bits/min) by 47.15 bits/min and 52.96 bits/min, and exceeding SSVEPNet (123.46 bits/min and 126.26 bits/min) by 41.29 bits/min and 39.33 bits/min separately. At the 1.2 s time window, although the ITR decreases to 158.60 bits/min slightly, it still outperforms FB-tCNN (107.17 bits/min), TDCA (110.71 bits/min), and TRCA (87.76 bits/min) by 51.43 bits/min, 47.89 bits/min and 70.84 bits/min dramatically, reflecting superior information transmission capability.

### 4.3 Results on Dataset C

We took advantage of LOSOCV as well. Tables 8, 9 display the classification accuracy and ITR of different methods under different time windows. It is undeniable that SSVEP-TFFNet still performs best. Under the 0.8 s time window, our method achieves an accuracy of 70.76% which is 15.57 and 32.52% higher than TDCA (55.19%) and FB-tCNN (38.24%), respectively. As the window increases to 1.0 s and 1.2 s, the accuracy rises to 77.37 and 82.50%. Under the 1.2 s time window, our method surpasses CCNN (75.61%) by 6.89% and EEGNet (74.55%) by 7.95%, revealing better performance. In the light of ITR, our method performs best as well across all time windows. For the 0.8 s and 1.0 s windows, the ITR reaches 228.95bits/min and 210.86 bits/min, exceeding FBtCNN (91.78 bits/min and 123.48 bits/min) by 137.17 bits/min and 87.38 bits/min separately. At the 1.2 s time window, although the ITR decreases to 193.18 bits/min slightly, it still outperforms FB-tCNN (114.94 bits/min) and TDCA (145.79 bits/min) by 78.24 bits/min and 47.39 bits/min dramatically, reflecting superior information transmission capability.

**Table 8:** Mean classification accuracy (%) across subjects for different methods under different time window lengths on Dataset C.

Method	Length of time (s)		
	0.8	1.0	1.2
TRCA	46.73 ± 26.44	54.37 ± 27.76	57.48 ± 27.71
TDCA	55.19 ± 21.24	64.33 ± 21.49	68.68 ± 20.43
EEGNet	63.71 ± 20.95	70.56 ± 21.04	74.55 ± 20.57
CCNN	62.30 ± 21.49	71.19 ± 20.20	75.61 ± 21.22
FBtCNN	38.24 ± 19.59	54.08 ± 22.40	58.23 ± 22.72
Ours	70.76 ± 20.25	77.37 ± 19.91	82.50 ± 17.24

**Table 9:** Mean ITR (bits/min) across subjects for different methods under varying time window lengths on Dataset C.

Method	Length of time (s)		
	0.8	1.0	1.2
TRCA	129.88 ± 103.48	129.91 ± 91.99	117.11 ± 78.60
TDCA	157.70 ± 88.60	159.09 ± 75.89	145.79 ± 62.28
EEGNet	195.27 ± 91.75	182.91 ± 76.89	165.95 ± 65.13
CCNN	189.30 ± 92.85	184.70 ± 74.51	170.21 ± 66.61
FBtCNN	91.78 ± 69.15	123.48 ± 72.86	114.94 ± 63.45
Ours	228.95 ± 93.21	210.86 ± 77.34	193.18 ± 58.15

## 5 Discussion

Results indicate that shorter time windows exist significant merits in high ITR applications, while longer windows are more suitable in high classification accuracy scenarios. Our proposed method captures multi-channel spatial features and fuses temporal and spectral characteristics effectively. It outperforms classical methods (FBCCA and TRCA) and leading deep learning models (EEGNet, CCNN, FB-tCNN, and SSVEPNet) in classification accuracy consistently, while also achieving superior ITRs. To better understand the proposed model and assess its potential applications, we consider and discuss three key factors. Firstly, in view of the portability and computational complexity, we varied the number of channels and evaluated the model’s performance. Secondly, we performed an ablation study to assess the contribution of each module quantitatively. Third, we utilized visualization techniques to reveal the distinctiveness of the features, thereby enhancing the model’s interpretability during decision-making process.

### 5.1 The influence of the number of channels

**Figure 2:**
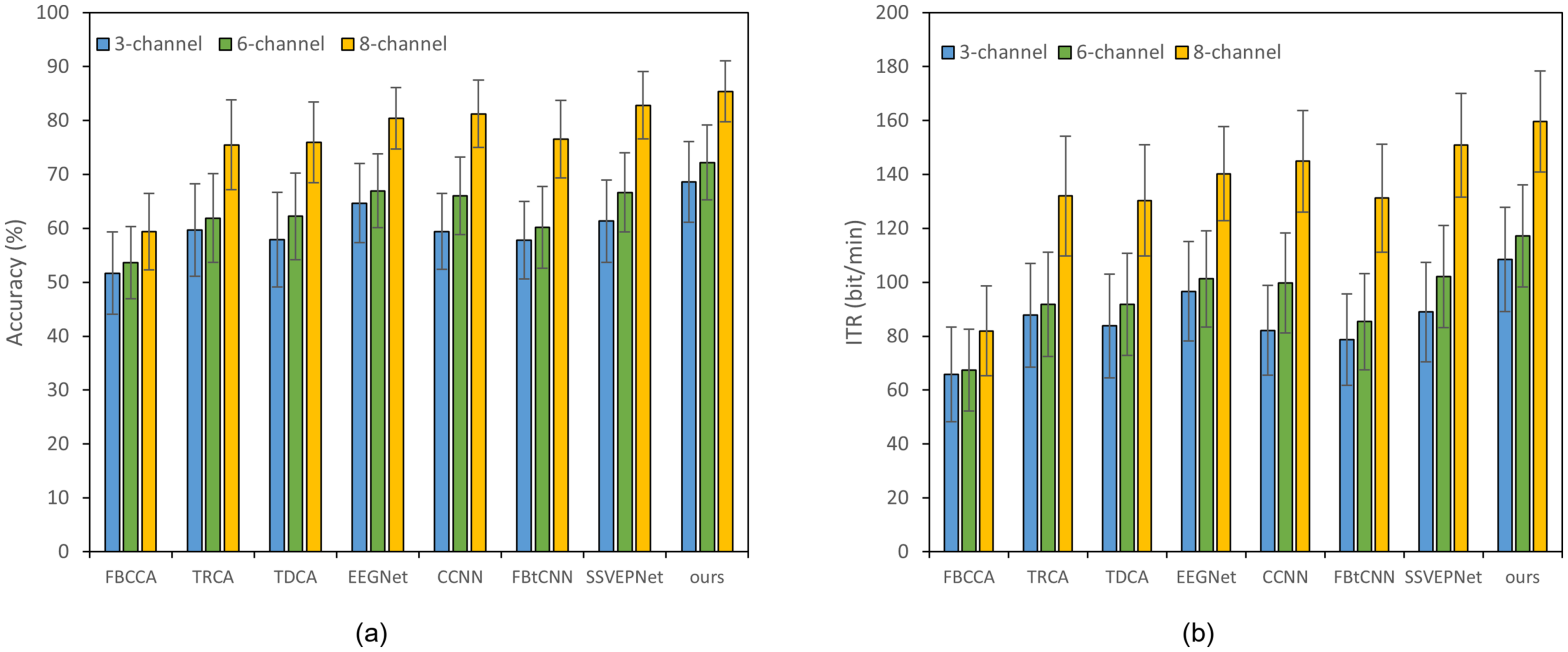
Classification accuracy and ITR of various methods on Dataset A with a 1.0 s time window under different channels; error bars represent the standard deviations. **(a)** Classification accuracy. **(b)** ITR.

**Figure 3:**
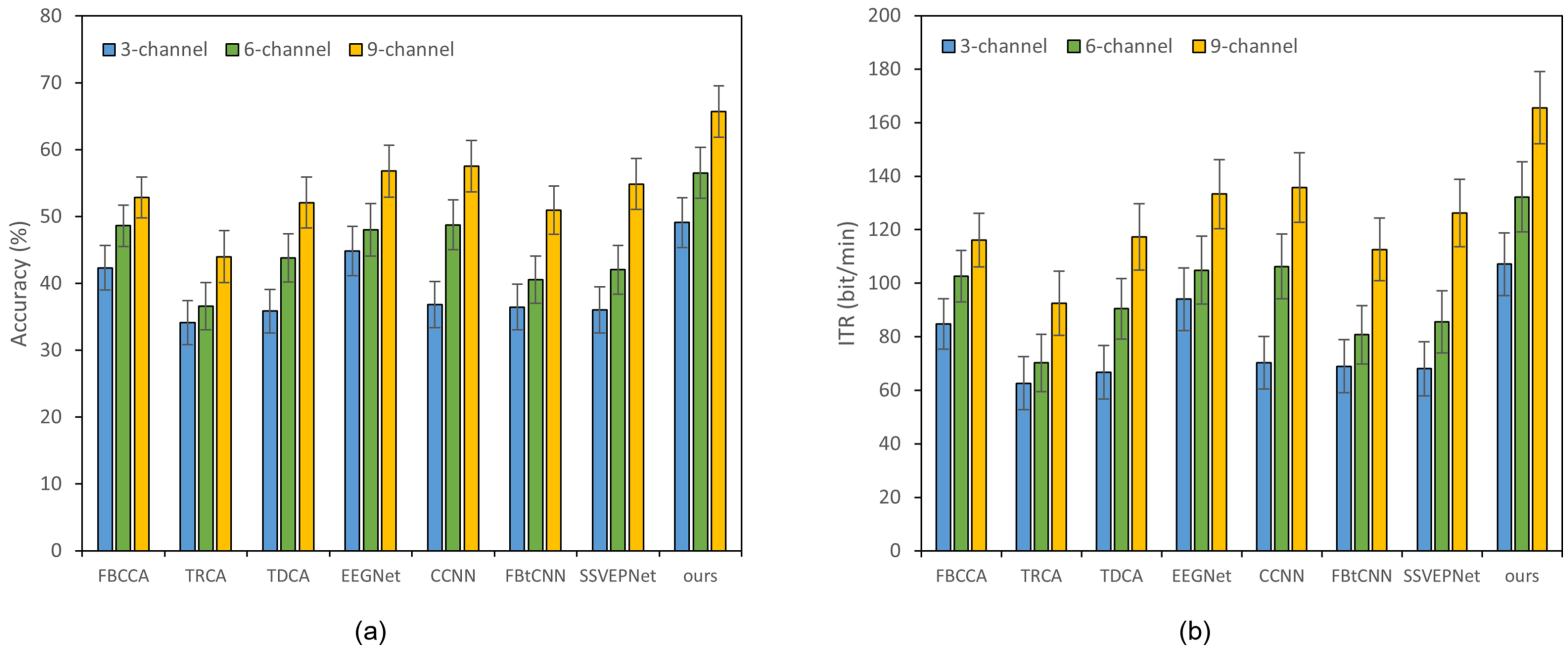
Classification accuracy and ITR of various methods on Dataset B with a 1.0 s time window under different channels; error bars represent the standard deviations. **(a)** Classification accuracy. **(b)** ITR.

Reducing the number of EEG channel plays an essential role for portable devices, which not only simplifies the configuration procedure but also improves wearing comfort (Minguillon et al., 2017). Meanwhile, it reduces the learning cost of users and enhances user experience (Craik et al., 2023). Figures 2, 3 illustrate the impact of different channel numbers (Dataset A: 3, 6, and 8 channels; Dataset B: 3, 6, and 9 channels) on the classification accuracy and ITR of each method under a fixed time window length (1.0 s). The exact numerical results are given in Supplementary material. Thereinto, Dataset B was chosen as an example of 40-class dataset. The results show that as the channel number increases, both the accuracy and ITR of all methods rise, indicating that more channels bring richer spatial features and thus better the performance of frequency recognition. More importantly, our proposed dual-branch network outperforms all controlled methods under all channel numbers. Even if we select data from only 3 channels, our proposed method maintains superior performance. Taking Dataset A as an example, SSVEP-TFFNet achieves an accuracy of 68.61% which is 8.89 and 7.28% higher than that of TRCA (59.72%) and SSVEPNet (61.33%), respectively. Notably, the performance of SSVEP-TFFNet with only 3 channels exceeds that of other methods using 6 channels. In Dataset A, it outperforms TRCA (61.89%) and SSVEPNet (66.67%) with 6 channels. In Dataset B, it surpasses CCNN (48.77%) and EEGNet (48.02%) with 6 channels. The findings reveal the preferable feature expression capability of our dual-branch structure under channel-limited scenarios, demonstrating its practicability in different channel configurations.

### 5.2 Ablation analysis

**Figure 4:**
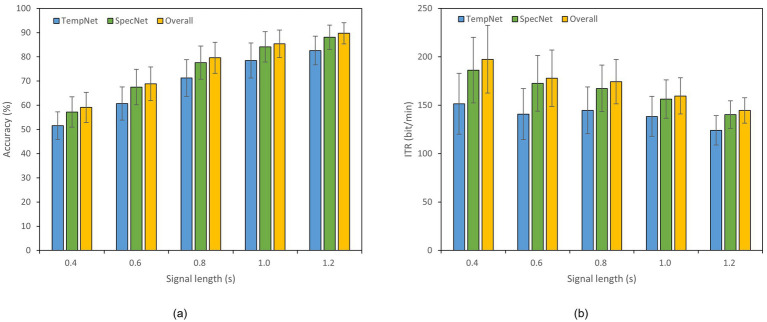
Results of the ablation study on Dataset A. The x-axis indicates the time window lengths, and the error bars represent the standard error. **(a)** Classification accuracy. **(b)** ITR.

**Figure 5:**
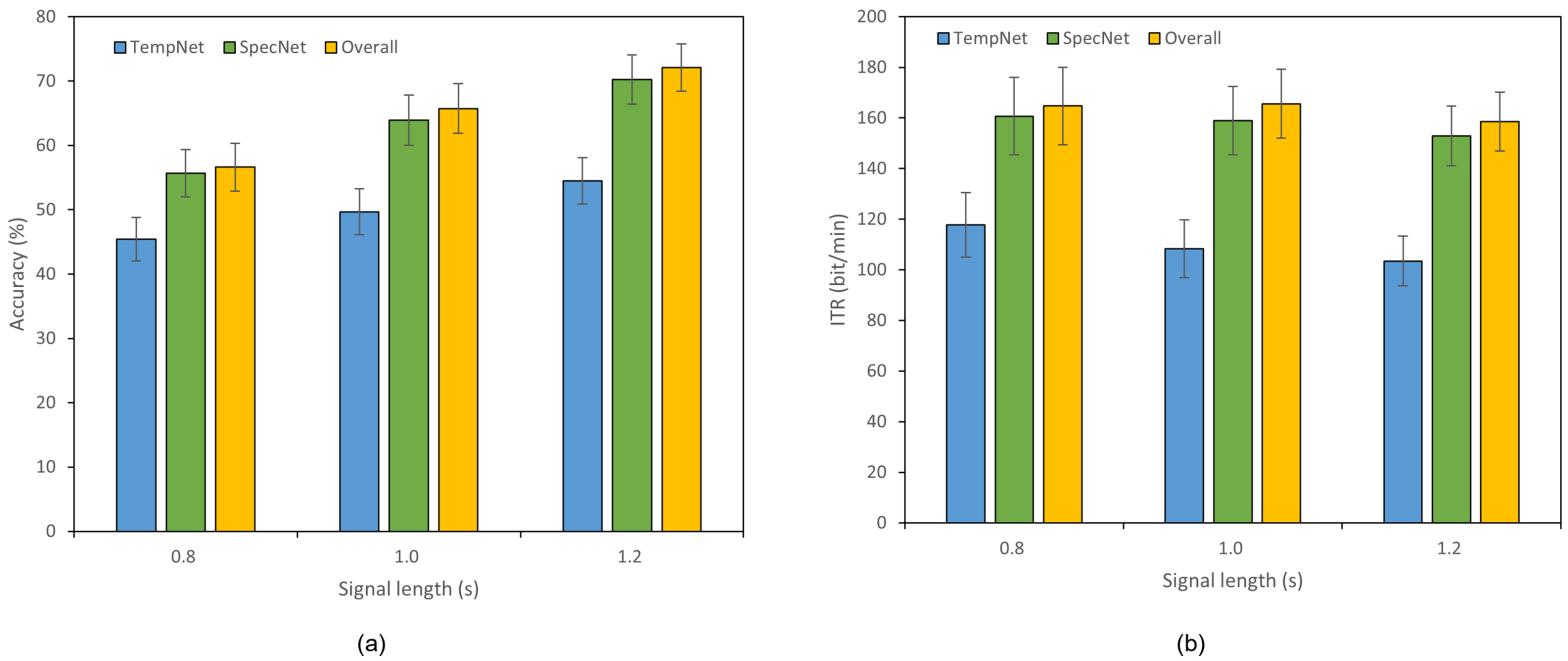
Results of the ablation study on Dataset B. The x-axis indicates the time window lengths, and the error bars represent the standard error. **(a)** Classification accuracy. **(b)** ITR.

To evaluate the effectiveness of our dual-branch feature fusion structure, we conducted ablation studies on Datasets A and B. Thereof, Dataset B was selected as an example of 40-class dataset. Specifically, we compared the classification accuracy and ITR across three models: (1) the model incorporating only time-domain feature extraction branch, (2) the model utilizing only frequency-domain feature extraction branch, and (3) the complete model with both branches. The experiments were performed under different time window lengths, as displayed in Figures 4, 5. In Dataset A, the accuracy of the complete model under a 0.4 s time window is 59.11% which is 7.55% higher than that of the model with only time domain feature extraction branch. The overall accuracy is also 1.89% higher than the accuracy of the model with only frequency domain feature extraction branch. In a 0.8 s window, the accuracy of the complete model reaches 79.61% which is 8.33% higher than that of the time domain feature extraction branch and 2.00% higher than that of the frequency domain feature extraction branch. In Dataset B, the accuracy of the complete model under a 1.2 s window is 72.11% which is 17.61% higher than that of the model with only time domain feature extraction branch. The overall accuracy is 1.86% higher than the accuracy of the model with only frequency domain feature extraction branch as well. These results reveal that our dual-feature extraction branch fusion structure is competent to learn and fuse time domain and frequency domain features adaptively, enhancing the expression of signals and suppressing noise effectively. In this case, the classification accuracy and robustness of the model are able to be improved considerably in cross-subject SSVEP decoding task (Figures 4, 5).

### 5.3 Feature visualization analysis

**Figure 6:**
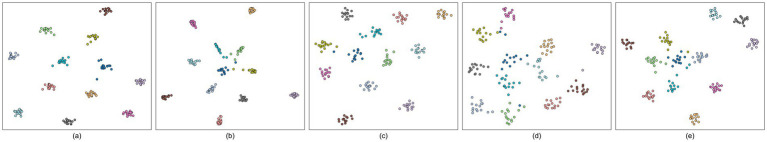
t-SNE visualizations of five models for #Subject 8 on Dataset A. Each scatter corresponds to one trial, and different colors different classes. **(a)** SSVEP-TFFNet. **(b)** SSVEPNet. **(c)** CCNN. **(d)** FB-tCNN. **(e)** EEGNet.

We employed t-distributed stochastic neighbor embedding (t-SNE) to visualize the high-level features extracted by deep learning model in two dimensions, thereby reflecting their distributions indirectly in raw high-dimensional space (Maaten and Hinton, 2008). Inasmuch as Dataset B and C contain relatively excessive target frequencies for clear visualization, the analysis was conducted on Dataset A only using a fixed 1 s time window. Taking Subject #8 as an example, the comparison of SSVEP-TFFNet with SSVEPNet, CCNN, FB-tCNN, and EEGNet are depicted in Figures 6a–e. Each scatter denotes one test trial. There are 12 frequency classes and each class is composed of 15 trials. The t-SNE map shows our method exhibits obvious intra-class cohesion and inter-class separation: samples from the same class cluster tightly, while different classes are obviously isolated. By comparison, SSVEPNet achieves intra-class compactness but many scatters of different classes converge towards the center, indicating poor separation. FB-tCNN and EEGNet both illustrate intra-class dispersion and insufficient inter-class distance. CCNN demonstrates clear inter-class isolation but displays loose intra-class distribution.

**Figure 7:**
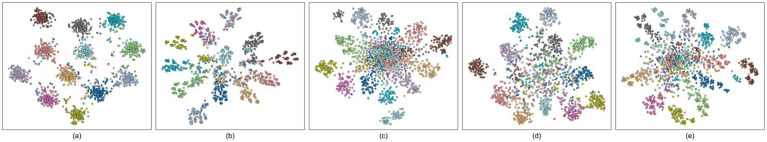
t-SNE visualizations of five models across all subjects on Dataset A. Each scatter represent to one trial, and different colors are on behalf of different classes. **(a)** SSVEP-TFFNet. **(b)** SSVEPNet. **(c)** CCNN. **(d)** FB-tCNN. **(e)** EEGNet.

We further aggregated the features of all 10 subjects (10 × 15 = 150 samples per class) and displayed the overall t-SNE visualization results in Figure 7. The results imply that SSVEP-TFFNet still maintains clear intra-class aggregation and inter-class separation, and different classes are distributed independently in low-dimensional space. The category boundaries of SSVEPNet are further cluttered. EEGNet, CCNN and FB-tCNN all express a large overlap of features of different categories. The above comparison further verifies the discriminatory ability of our dual-branch network in cross-subject SSVEP frequency recognition, providing an intuitive explanation for its superior performance.

### 5.4 Limitations

In Dataset B, we analyzed the first 35 subjects determined by dataset index. While this selection rule avoids any performance-driven selection bias and ensures reproducibility, it may potentially introduce a minor order-related bias if the publication order of subjects is correlated with hidden subject characteristics (such as age, gender, attention level, etc.). Therefore, the current results is interpreted as evidence on the fixed and reproducible 35-subject subset. Nevertheless, this limitation does not undermine our methodology. Our proposed approach demonstrates superior performance under the LOSO evaluation protocol.

## 6 Conclusion

To address the challenges of high data acquisition costs, limited cross-subject generalization, and the reliance of principal methods on single-domain features, we propose SSVEP-TFFNet, a dual-branch feature fusion network for SSVEP frequency recognition. This model extracts discriminative features from time and frequency domains independently and fuses them adaptively via a dynamic weighting mechanism, enhancing feature representation significantly. Evaluations on three public datasets demonstrate that SSVEP-TFFNet outperforms both traditional algorithms and mainstream deep learning models consistently in cross-subject classification accuracy and ITR, without requiring any subject-specific calibration. Furthermore, the model achieves relatively high recognition rates even with minimal channels. Ablation studies verify the efficacy of the dual-branch fusion mechanism, while feature visualizations offer intuitive explanation into its superior discriminability. The characteristics of calibration-free and cross-subject lower the deployment threshold and usage cost of real-world BCI system. Effective channel selection enhances the portability of EEG acquisition devices and improves the wearing comfort. These are very beneficial for immersive operations in virtual reality (VR) environments or long-term use in daily assistive technologies.

## Data Availability

The original contributions presented in the study are included in the article/Supplementary material, further inquiries can be directed to the corresponding author.
