# Supplementary material for: A time-frequency feature fusion-based deep learning network for SSVEP frequency recognition
Source: Front Neurosci. 2025 Sep 29;19:1679451. doi: 10.3389/fnins.2025.1679451 (PMC12515880; doi:10.3389/fnins.2025.1679451)
Supplement: Supplementary file 1 [file Table_1.docx]

**Supplementary material**

Table 1. Mean classification accuracy (%) across subjects for different methods under different number of EEG channels on Dataset A.

| Method | Number of EEG Channels | | |
| --- | --- | --- | --- |
|  | 3 | 6 | 8 |
| FBCCA | 51.67±24.13 | 53.67±21.19 | 59.39±22.50 |
| TRCA | 59.72±27.11 | 61.89±26.11 | 75.50±26.48 |
| TDCA | 57.89±27.75 | 62.22±25.32 | 75.94±23.77 |
| EEGNet | 64.67±23.22 | 66.94±21.64 | 80.39±18.10 |
| CCNN | 59.39±22.27 | 66.00±22.72 | 81.22±19.80 |
| FBtCNN | 57.78±22.77 | 60.17±24.07 | 76.50±22.75 |
| SSVEPNet | 61.33±24.23 | 66.67±23.30 | 82.83±19.65 |
| Ours | 68.61±23.76 | 72.22±22.09 | 85.39±17.90 |

Table 2. Mean ITR (bits/min) across subjects for different methods under different number of EEG channels on Dataset A.

| Method | Number of EEG Channels | | |
| --- | --- | --- | --- |
|  | 3 | 6 | 8 |
| FBCCA | 65.87±55.41 | 67.40±48.02 | 81.99±52.73 |
| TRCA | 87.76±60.93 | 91.77±61.25 | 132.01±70.37 |
| TDCA | 83.81±60.71 | 91.86±59.84 | 130.38±65.38 |
| EEGNet | 96.59±58.29 | 101.26±56.58 | 140.31±55.38 |
| CCNN | 82.16±52.85 | 99.74±58.80 | 144.91±59.49 |
| FBtCNN | 78.71±53.65 | 85.38±56.53 | 131.20±63.43 |
| SSVEPNet | 88.98±58.29 | 102.09±59.71 | 150.85±60.86 |
| Ours | 108.39±61.15 | 117.19±59.92 | 159.61±59.05 |

Table 3. Mean classification accuracy (%) across subjects for different methods under different number of EEG channels on Dataset B.

| Method | Number of EEG Channels | | |
| --- | --- | --- | --- |
|  | 3 | 6 | 9 |
| FBCCA | 42.32±19.64 | 48.64±18.28 | 52.88±18.12 |
| TRCA | 34.14±19.62 | 36.61±20.95 | 44.00±22.90 |
| TDCA | 35.86±19.17 | 43.79±21.38 | 52.09±22.55 |
| EEGNet | 44.86±21.82 | 48.02±23.18 | 56.79±22.98 |
| CCNN | 36.80±20.39 | 48.77±22.19 | 57.55±22.82 |
| FBtCNN | 36.45±20.13 | 40.57±20.87 | 50.95±21.54 |
| SSVEPNet | 36.02±20.39 | 42.04±21.63 | 54.86±22.53 |
| Ours | 49.09±21.92 | 56.54±22.72 | 65.73±22.84 |

Table 4. Mean ITR (bits/min) across subjects for different methods under different number of EEG channels on Dataset B.

| Method | Number of EEG Channels | | |
| --- | --- | --- | --- |
|  | 3 | 6 | 9 |
| FBCCA | 84.81±55.70 | 102.68±56.87 | 116.14±58.96 |
| TRCA | 62.66±59.22 | 70.24±63.13 | 92.51±71.25 |
| TDCA | 66.64±59.21 | 90.47±67.18 | 117.24±73.36 |
| EEGNet | 94.01±68.74 | 104.82±75.10 | 133.30±76.50 |
| CCNN | 70.24±58.04 | 106.24±71.56 | 135.73±76.45 |
| FBtCNN | 69.01±58.78 | 80.79±64.51 | 112.63±69.51 |
| SSVEPNet | 68.04±60.01 | 85.62±68.44 | 126.26±74.75 |
| Ours | 107.12±69.31 | 132.22±77.35 | 165.59±80.32 |
